# The Benefit Finding Questionnaire (BFQ): Scale Development, Validation, and Its Psychometric Properties Among People with Mental Illness

**DOI:** 10.3390/healthcare8030303

**Published:** 2020-08-26

**Authors:** Rie Chiba, Akiko Funakoshi, Yoshihiko Yamazaki, Yuki Miyamoto

**Affiliations:** 1 Department of Nursing, Graduate School of Health Sciences, Kobe University, Kobe 654-0142, Japan; 2 College of Nursing, Kobe City College of Nursing, Kobe 651-2103, Japan; akiko-funakoshi@umin.ac.jp; 3 Faculty of Social Welfare, Nihon Fukushi University, Aichi 470-3295, Japan; yamazaki_1112@yahoo.co.jp; 4 Department of Psychiatric Nursing, Graduate School of Medicine, The University of Tokyo, Tokyo 113-0033, Japan; yyuki-tky@umin.ac.jp

**Keywords:** benefit finding, coping, Japan, mental illness, positive psychology, psychometrics, scale development, questionnaire, validation

## Abstract

Benefit finding has been defined as positive life changes that result from a stressful event, such as the diagnosis of chronic illness. The present study aimed to develop a benefit finding questionnaire (BFQ) and examine its psychometric property among people with chronic mental illness in Japan. This study adopted a mixed method composed of three phases, including Phase 1: To draft the item pool and design the BFQ based on literature review and discussion among the authors, Phase 2: To revise and refine the drafted items through feedback from focus group interviews and further consideration, and Phase 3: To examine the psychometric properties of the BFQ following the questionnaire survey for people with chronic mental illness and validation of the questionnaire. In Phase 3, a cross-sectional, self-administered questionnaire survey was conducted for mental health service users. Among the 373 eligible participants, we used data from 265 respondents for the analyses (valid response rate = 71.0%). About 65% were male, and the average age was 45.3 years (SD = 12.9). Around 70% were diagnosed with schizophrenia. Factorial, concurrent, and divergent validities, as well as reliability were explored. The 21-item BFQ demonstrated good factorial validity, concurrent and divergent validities, and sufficient internal consistency reliability among people with chronic mental illness. It appears to be a useful scale to assess experience of benefit finding among people with chronic mental illness. Further large-scale research will ensure verification of the scale among people with other illnesses or difficulties.

## 1. Introduction

While psychiatry has traditionally focused on psychopathology and treating mental illnesses to alleviate symptoms and prevent relapse, positive psychiatry has come to the forefront over recent years [1,2]. Positive psychiatry, aligned with its precedent, positive psychology, seeks to expand the scope of psychiatry to include broader aspects of mental health and well-being with core concepts such as personal recovery, resilience, optimism, and hope [1,2,3]. In this context, the potential for positive psychological changes following a negative event, such as chronic illness, has gained substantial attention [4].

Benefit finding, a concept that fits with positive psychiatry and positive psychology, has been defined as positive life changes that result from a stressful event such as the diagnosis of chronic illness [5]. Some researchers interpret benefit finding as a positively oriented emotional coping strategy in adversity [6,7]. In this light, benefit finding is described as a positive reappraisal process that can facilitate positive coping emotions and behaviors among people going through life-changing experiences [5,8]. In other words, benefit finding has also been conceptualized as a construction of positive meaning-making [9,10].

Several conceptualizations such as post-traumatic growth (PTG) [11] and stress-related growth [12] have also been suggested to describe positive changes following threatening events. Though these terms are often used interchangeably, they are conceptually different [13]. For example, benefit finding is conceptualized as a process trying to find positive changes or benefits through the experience of adversity [8,13]. On the other hand, PTG is conceptualized as an outcome which encompasses a totally transformative experience, resulting from a pervasive cognitive shift that is undergone by relatively few of those who report positive changes following adversity [13]. Since benefit finding is theoretically considered one of cognitive processes that lead to the development of PTG [13], it could be an important phenomenon which contributes to overall psychological growth. Personal recovery is also a concept that includes positive changes. It is described as a complex and subjective process of developing new meaning and purpose in life as people grow beyond the catastrophic effects of mental illness [14]. While personal recovery and benefit finding have similar perspectives, personal recovery focuses on people with mental illness, and it includes more comprehensive processes of their lives compared to benefit finding [15].

In the psychiatric field, an earlier qualitative study on benefit finding among Japanese people with mental illness revealed several themes related to strengthened relationship with others, personal change of values in life, health-related behavioral changes, increased understanding of mental illness, and finding a new role in society as answers in small numbers [16]. Previous studies also indicated that understanding the experience of benefit finding would contribute to the support of further progress in personal recovery among people with mental illness [15,17].

To date, many researchers have suggested assessing the subjective experience of benefit finding, and many scales have been developed indeed [7,8]. However, most of these scales were developed for use in patients with chronic physical illnesses such as cancer [8]; no such scale applicable for people with mental illness has been developed or examined. Mental illness goes chronically with repeated improvement and deterioration, while physical illness often causes life threats. Since benefit finding among people with mental illness has been studied in recent years [15,16,17,18], the scale appropriate for people with mental illness is also needed. Furthermore, though the number of studies on benefit finding among people with chronic illness in Asian countries has been increasing recently [19,20,21,22,23], most scales to assess benefit finding were developed in Western countries [24]. Since the aspects of benefit finding may be different between Asian countries and Western countries [19,21], whether such existing scales are sufficiently applicable in Asian countries has yet to be elucidated. Thus, this study aimed to develop a scale to assess benefit finding in Japan and to examine its psychometric properties among people with chronic mental illness.

## 2. Methods

### 2.1. Three Phases of the Study Procedure

This study adopted a mixed method composed of three phases.

**Phase 1**: We drafted the item pool and designed the benefit finding questionnaire (BFQ) based on literature review and discussion among the authors.
**Phase 2**: We revised and refined the drafted items through feedback from focus group interviews and further consideration.
**Phase 3**: The psychometric properties of the BFQ were examined following the questionnaire survey for people with chronic mental illness and validation of the questionnaire.

### 2.2. Phase 1: The Procedure of the Item Pool and Designing the BFQ

Based on earlier qualitative studies on benefit finding in people with chronic mental illness and those of physical illness in Japan [16,25], we pooled items and included them in a draft of the initial questionnaire through discussions among the authors. Literature review of scale development for benefit finding and other relevant concepts was also conducted and referenced in designing the questionnaire.

### 2.3. Phase 2: Revision and Refinement of the Drafted Items Through Focus Group Interviews

Two focus group interviews were conducted using the same four people with chronic mental illness at a welfare facility in a community in the Kanto area in Japan. First, they were asked to discuss the face validity and understandability of each candidate item as well as semantically overlapping or resembling items of the initial questionnaire. On a later day, the second focus group interview was held, and participants were asked to offer comments about the methods of querying, including the introductory sentences and the volume of the initial questionnaire.

### 2.4. Phase 3: Questionnaire Survey, Validation, and Testing Its Psychometric Properties

#### 2.4.1. Participants

A cross-sectional, self-administered questionnaire survey was conducted for mental health service users from August to September 2014. The following were eligible for inclusion: (1) diagnosed with mental illness by a psychiatrist, (2) aged 20 or older, (3) living in the community, (4) service user of the facility for people with mental illness, and (5) not diagnosed with mental retardation or dementia. We conducted the survey in six psychiatric daycare centers of six hospitals in the Kanto region as well as in sixteen facilities of six incorporated non-profit organizations in Kanto, Japan. A total of 295 of the 373 eligible participants agreed to participate in the study and returned the questionnaire. Those who had at least one missing value on the BFQ and who did not meet all the inclusion criteria were excluded (*n* = 30). Thus, the data from the remaining 265 respondents were used for the analyses (valid response rate = 71.0%).

#### 2.4.2. Measures

##### Benefit Finding Questionnaire (BFQ)

The initial 30-item BFQ was included in the questionnaire. All items are rated on a 5-point Likert scale, and higher total scores indicate further experiences of benefit finding.

##### Recovery Assessment Scale (RAS)

The RAS assesses personal recovery among people with mental illness [26,27]. Factor analyses in a Japanese study revealed five factors: goal/success orientation and hope, reliance on others, personal confidence, no domination by symptoms, and willingness to ask for help [28]. Items are rated on a 5-point Likert scale, and higher total scores indicate further progress in recovery. Its reliability and validity of the Japanese version have been confirmed (Cronbach’s alpha coefficient = 0.89) [28].

##### Self-Identified Stage of Recovery Part-A

Andresen, Capti, and Oades (2010) [29] developed the five-stage recovery model among people with mental illness, including moratorium, awareness, preparation, rebuilding, and growth. The Self-Identified Stage of Recovery Part-A (SISR-A) is a single-item, forced-choice measure selecting the most applicable of the five statements representing each stage of personal recovery [29]. A fair level of test–retest reliability and good concurrent validity of the Japanese version have been demonstrated by a questionnaire survey for people with chronic mental illness [30].

##### Self-Identified Stage of Recovery Part-B

The Self-Identified Stage of Recovery Part-B (SISR-B) consists of four items assessing the following key component processes of personal recovery: “finding hope”, “re-establishment of identity”, “finding meaning”, and “taking responsibility” [29]. These four items are rated on a 6-point Likert scale. A higher total score indicates a higher level of recovery. Good internal consistency, reasonable test–retest reliability, and good concurrent reliability of the Japanese version have been demonstrated (Cronbach’s alpha coefficient = 0.80) [30].

##### The Herth Hope Index

The Herth Hope Index (HHI) is a scale assessing one’s level of hope [31]. Items are rated on a 4-point Likert scale; a higher score indicates a higher level of hope. The reliability and validity of the Japanese version have been confirmed (Cronbach’s alpha coefficient = 0.89) [32].

##### The Short-Form Health Survey (SF-8)

Quality of life (QOL) was assessed using the standard version of the SF-8 [33], an 8-item scale that provides physical and mental component summary scores with higher scores indicating better QOL. The reliability and validity of the Japanese version have been confirmed [34].

#### 2.4.3. Statistical Analysis

We first conducted preliminary analysis and considered the items that should be eliminated in terms of resemblance, lack of clarity, and unstable factor structure. After eliminating such items, the suitability of the data for factor analysis was examined using the Kaiser–Mayer–Olkin (KMO) measure of sampling adequacy together with Bartlett’s chi-square test of sphericity. The KMO indicator was then compared with adequacy standards (marvelous, >0.90) [35]. To extract a factor structure of the scale items to address factorial validity, explanatory factor analysis (EFA) was performed using the unweighted least squares method and promax rotation. The number of the factors was determined by the eigen values with 1 or more. Following this, confirmatory factor analysis (CFA) was employed to test the model fit of the data to the factor structure extracted from EFA. Model fit was assessed using a combination of fit indices, including the comparative fit index (CFI), Tucker–Lewis Index (TLI), and root mean square error of approximation (RMSEA) in accordance with the COSMIN (COnsensus-based Standards for the selection of health Measurement INstruments) methodology [36]. The acceptability of model fit was judged by the recommended criteria with CFI and TLI > 0.90, RMSEA < 0.06 [36]. Theoretically, CFA is suggested to be conducted on a separate sample to confirm the structure of the proposed scale resulting from an EFA [37]. On the other hand, a sample of at least 200–300 or minimum of 10 participants for each item for EFA have been suggested to reduce the error rate [37,38,39,40]. Given such recommendations, we used the same whole sample for EFA and CFA in this study, in reference to earlier studies which conducted their analyses in the same manner [41].

Concurrent and divergent validities were examined by calculating Pearson’s or Spearman’s correlation coefficients between the total BFQ score and the scores on the other five scales. We hypothesized that personal recovery and hope would strongly and positively correlate with benefit finding, whereas mental and physical health would not be strongly related with benefit finding.

Cronbach’s alpha coefficients per subscale of BFQ were calculated to evaluate the internal consistency reliability. A score above 0.70 was taken to indicate sufficient homogeneity of the items [36]. Test–retest reliability was evaluated after two weeks in 19 participants. This time interval between test and retest was chosen because we considered it to be long enough to prevent recall of previous answer but short enough to assume that the perception would not change in most cases. The intraclass correlation coefficient (ICC) for each domain and the quadratic weighted kappa value of each item were calculated. Both ICC and weighted kappa should be above 0.70 [36].

All statistical analyses, except for CFA, were conducted using SPSS 25.0J (IBM, New York, NY, USA) for Windows; CFA was conducted using Amos ver. 25.0 (IBM, New York, NY, USA). Values of *p* less than 0.05 were considered statistically significant (two-tailed tests).

#### 2.4.4. Ethical Considerations

This study was approved by the ethical committee of Jichi Medical University (EKI 14-27), to which the first author belonged. All participants received oral explanations with written documentation about the purpose and methods. Providing an answer represented their agreement to participate in the study. The survey was conducted anonymously.

## 3. Results

### 3.1. Phase 1

Based on the literature review, we pooled 30 items reflecting benefit finding in the experience of chronic illness including mental illness and put them into the initial BFQ. The adequacy of each item to assess benefit finding was evaluated by researchers in psychiatric nursing and health sociology. According to a qualitative study among people with chronic mental illness in Japan, about 80% of benefit finding was consolidated into two categories, i.e., strengthened relationship with others and personal change of values in life. The former category included themes such as awareness of others’ tenderness, sympathy toward others, and so on. The latter category involved themes such as inner strength, cherishing oneself, and so on [16]. Thus, we hypothesized an appropriate factor structure was considered to be two.

The literature review revealed that there are scales of benefit finding that assess only one’s positive changes [42,43], as well as scales that assess both positive and negative changes [44,45,46,47]. In this study, some items assess only positive changes, while others ask about positive and negative changes according to the content of the items.

### 3.2. Phase 2

The face validity and understandability of each item was confirmed by the focus group interview. Based on the feedback, some items were revised or refined to be more understandable and clearer. The method of querying, including the introductory sentences and volume, was checked through the focus group interview. Semantically resembling items remained whole without elimination at that point.

### 3.3. Phase 3

In Phase 3, we conducted the questionnaire survey and examined its properties.

#### 3.3.1. Validation of BFQ

No items had a ceiling or floor effect, while normality in each item was not supported by the Kolmogorov–Smirnov test (*p* < 0.01, respectively). All 30 items lacked the acceptable factor structure. Thus, we carefully decided to blank nine items that were semasiologically similar to other items or those that left a sense of indistinctness following cautious discussion. We used the remaining 21 items for further analyses (Supplementary Files: A for English version, B for Japanese version). The total scores on the 21-item BFQ showed normality by the Kolmogorov–Smirnov test (*p* = 0.2)

#### 3.3.2. Characteristics of Respondents

Table 1 shows the sociodemographic and clinical characteristics of the respondents. Respondents were aged between 20 and 76 years (mean = 45.3, standard deviation = 12.9), and about 65% were male. Around 70% were diagnosed with schizophrenia. Three-quarters had experienced hospitalization in psychiatric wards.

#### 3.3.3. Factorial Validity

**Figure 1:**
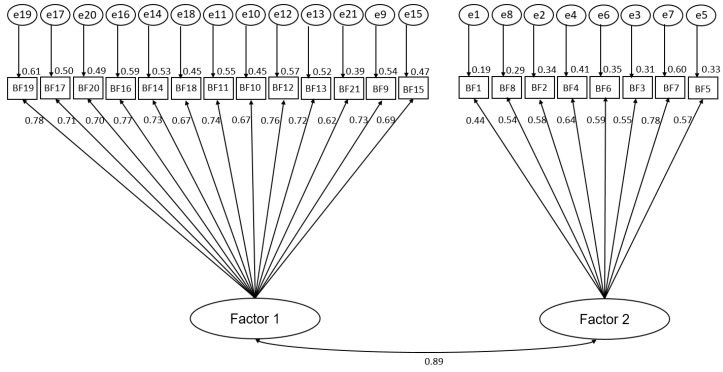
Path diagram of BFQ, showing standardized coefficients from confirmatory factor analysis (*N* = 265). Factor 1: Changes in sense of values and way of thinking; Factor 2: Changes in relationships with others.

The KMO was 0.95, indicating good sampling adequacy with significance on Bartlett’s test of sphericity (χ^2^ = 2771.0, *df* = 210, *p* < 0.001). Among the 265 respondents, EFA yielded two factors (Table 2). The two extracted domains were considered to represent “Changes in sense of values and way of thinking” and “Changes in relationships with others”. CFA indicated that this extracted factor structure fit the data completely (CFI = 0.94; TLI = 0.94; RMSEA = 0.055) (Figure 1).

#### 3.3.4. Concurrent and Divergent Validity

Table 3 shows that the total BFQ score was significantly and positively correlated with the total scores on the RAS (*r* = 0.84; *p* < 0.01), SISR-A (*ρ* = 0.47; *p* < 0.01), SISR-B (*r* = 0.72; *p* < 0.01), and HHI (*r* = 0.72; *p* < 0.01). Weak but significant and positive correlations were found between the total scores on the BFQ and SF-8 (mental component) (*r* = 0.23; *p* < 0.01), as well as SF-8 (physical component) (*r* = 0.19; *p* < 0.01).

#### 3.3.5. Reliability (Internal Consistency Reliability and Test–Retest Reliability) 

Cronbach’s alpha coefficients were 0.93 for Factor 1 and 0.81 for Factor 2, indicating sufficient homogeneity of all items in the subscales. The ICCs were 0.91 for Factor 1 and 0.96 for Factor 2, indicating good reliability. Regarding the kappa values for each item, 12 out of 21 items had weighted kappa values above 0.70, indicating excellent reliability, while the other nine items (#1, 2, 4, 8, 10, 12, 13, 17, 21) did not (Table 2).

## 4. Discussion

The 21-item BFQ developed in this study showed good factorial, concurrent, and divergent validity among people with chronic mental illness in a community in Japan. It also revealed good internal consistency and test–retest reliability in general, though some items did not show strong test–retest reliability. Normality in each item was not supported in this study. Some items such as “Your compassion and empathy for people have been…” and “Finding happiness in small things has become …” showed higher average scores. On the other hand, other items such as “A sense that you have become capable of feeling your hope and future has been gained …” and “Trustworthy friends or peers you would not have met if you did not have mental illness have been gained …” indicated lower average scores. This means that people with mental illness are more likely to experience some kinds of benefit finding, but less other kinds of benefit finding. Thus, non-normal distributions in each item do not mean that BFQ showed insufficient validity.

Two factors, “Changes in sense of values and way of thinking” and “Changes in relationships with others”, are in common with those in earlier studies among people with chronic physical illness [43,47,48,49]. On the other hand, while earlier studies among people with physical illness revealed factors of health-related behavioral changes [43,47,49], such factors were not extracted in this study. While the item related to health behavior is also in the BFQ, it was consolidated to Factor 1 “Changes in sense of values and way of thinking” in this study. Health-related behavior in people with mental illness may differ from that in people with physical illness. Given the above, the BFQ seems to comprehensively capture positive changes common to both patients with mental illness and those with physical illness, but does not include specific health-related behavioral changes as a domain. Future research may reveal its applicability among people with a wide range of physical chronic illness and other difficulties. Earlier studies in Western countries revealed factors related to spirituality or faith [43,47]. While an even earlier study among patients with cancer in Japan also showed the factor of “faith” [42], this study did not generate such factors. Faith and spirituality in Japanese people with chronic illness may be a future issue to examine.

BFQ score was significantly and positively correlated with RAS, SISR-A, SISR-B, and HHI scores with weak and positive correlation with SF-8 (Mental) and SF-8 (Physical) scores. Since earlier studies revealed a positive relationship between benefit finding and not only psychological well-being but also subjective physical health [50], these relations were consistent with our hypotheses. Thus, this result showed good concurrent and divergent validities.

While the internal consistency was satisfactory, the quadratic weighted kappa values showed that some items lacked excellent test–retest reliability. Items such as ties with friends may change through impactful experiences even over a short period.

The BFQ included some items asking about positive and negative changes and other items asking only about positive changes, according to the content of the items. It has been suggested that including both positive and negative effects of illness in the same instrument provides a more valid assessment of benefit finding, reducing response bias [24,44,51]. Thus, the BFQ can be a good scale to avoid respondents’ psychological burden and social desirability bias.

### Limitations

Some limitations should be addressed. First, there were some non-respondents and some who might be more likely to have severe psychiatric symptoms or lower benefit finding. Therefore, the current average score of the BFQ in this study may be slightly overestimated. Furthermore, most of participants in this study were diagnosed with schizophrenia and had experienced hospitalization. Thus, the findings should be generalized with caution. Second, EFA and CFA were performed in the same sample in this study. To verify the dimensionality of the scale more rigorously, analyses in the different sample should be implemented in the future study. Further large-scale research is needed among a more diverse population of mental health service users to ascertain robust verification, particularly in terms of the test–retest reliability of the BFQ.

## 5. Conclusions

The present study developed a scale to assess benefit finding and examined its psychometric properties among people with chronic mental illness in Japan. The BFQ demonstrated good factorial validity, concurrent and divergent validities, and sufficient internal consistency reliability. It appears to be a useful scale to assess experience of benefit finding among people with chronic mental illness. Future research may reveal its applicability among people with a wide range of physical chronic illness and other difficulties.

## Figures and Tables

**Table 1:** Sociodemographic and clinical characteristics of the respondents in the study (*N* = 265).

Variables	Total		Male		Female	
	(*N* = 265)		(*n* = 173; 65.3%)		(*n* = 92; 34.7%)	
	*n* [Mean]	(%) [SD]	*n* [Mean]	(%) [SD]	*n* [Mean]	(%) [SD]
Age (years) (*n* = 263)	[45.3]	[12.9]	[45.9]	[12.6]	[44.2]	[13.4]
Duration of the illness (years) (*n* = 237)	[16.1]	[11.9]	[16.8]	[11.9]	[14.8]	[11.9]
Diagnosis						
Schizophrenia	187	(70.6)	123	(71.1)	64	(69.6)
Depression	24	(9.1)	17	(9.8)	7	(7.6)
Bipolar disorder	16	(6.0)	10	(5.8)	6	(6.5)
Others	31	(11.7)	19	(11.0)	12	(13.0)
Unknown	7	(2.6)	4	(2.3)	3	(3.3)
Coexisting physical illness						
Yes	102	(38.5)	73	(42.2)	29	(31.5)
No	156	(58.9)	99	(57.2)	57	(62.0)
Unknown	7	(2.6)	1	(0.6)	6	(6.5)
Experience of hospitalization for psychiatric wards						
Yes	209	(78.9)	139	(80.3)	70	(76.0)
No	36	(13.6)	25	(14.5)	11	(12.0)
Unknown	20	(7.5)	9	(5.2)	11	(12.0)
Recipient of Mental disability certificate						
Yes	169	(63.8)	105	(60.7)	64	(69.6)
No	96	(36.2)	68	(39.3)	28	(30.4)
Unknown	-		-		-	
Recipient of Disability pension						
Yes	162	(61.1)	107	(61.8)	55	(59.8)
No	103	(38.9)	66	(38.2)	37	(40.2)
Unknown	-		-		-	
Recipient of Livelihood protection						
Yes	71	(26.8)	46	(26.6)	25	(27.2)
No	194	(73.2)	127	(73.4)	67	(72.8)
Unknown	-		-		-	
Place of living						
Home of one’s own or apartment	206	(77.7)	134	(77.5)	72	(78.3)
Group-home	56	(21.1)	37	(21.4)	19	(20.7)
Unknown	3	(1.1)	2	(1.2)	1	(1.1)
Cohabitation (multiple answers allowed)						
With parents	122	(46.0)	75	(43.4)	47	(51.1)
With siblings	51	(19.2)	30	(17.3)	21	(22.8)
With a partner	10	(3.8)	6	(3.5)	4	(4.3)
With children	10	(3.8)	4	(2.3)	6	(6.5)
Single living	96	(36.2)	68	(39.3)	28	(30.4)

**Table 2:** Reliability and factors derived from the benefit finding questionnaire (BFQ) (*N* = 265): An item factor analysis with the unweighted least squares method and promax rotation.

No	Items	Weighted Kappa ^†^	Min	Max	Mean	SD	Skewness	Kurtosis	Factor	
									1	2
	**Factor 1: Changes in sense of values and way of thinking** **(Cronbach’s α = 0.93) (ICC ^†^ = 0.91)**									
19	A sense that you have become capable of feeling your hope and future has been gained …	0.76	1	5	2.60	1.23	0.37	−0.76	0.88	−0.11
17	A feeling that you are willing to begin something on your own has been …	0.65	1	5	3.18	1.12	−0.26	−0.41	0.87	−0.18
20	To work toward a healthy lifestyle that suits you has become …	0.80	1	5	3.33	1.13	−0.52	0.40	0.73	−0.03
16	New something to live for or enjoyment in life has been gained …	0.89	1	5	3.05	1.17	0.01	−0.65	0.73	0.06
14	A feeling that you are happy to be alive has been …	0.80	1	5	3.49	1.23	−0.57	−0.45	0.69	0.05
18	Your mental strength has been …	0.87	1	5	3.08	1.15	−0.24	−0.54	0.68	0.00
11	A sense that you are capable of facing your life with illness has been gained …	0.82	1	5	3.08	1.12	0.00	−0.57	0.66	0.10
10	A feeling that you cherish yourself have been …	0.51	1	5	3.49	1.09	−0.48	−0.29	0.65	0.04
12	A feeling that you got to focus on things you can do rather than things you cannot do has been gained …	0.51	1	5	3.03	1.08	0.01	−0.40	0.62	0.16
13	A sense that you would live by your own values rather than comparing yourself to others has been gained …	0.63	1	5	3.15	1.10	−0.14	−0.49	0.60	0.14
21	A feeling that you want to be of help to others and society has been …	0.42	1	5	3.49	1.04	−0.49	0.07	0.48	0.18
9	A feeling of reassurance knowing people who have similar illnesses or difficulties has been gained …	0.88	1	5	3.29	1.20	−0.26	−0.67	0.7	0.32
15	Finding happiness in small things has become …	0.74	1	5	3.55	1.10	−0.58	−0.17	0.45	0.28
	**Factor 2: Changes in relationships with others** **(Cronbach’s α = 0.81) (ICC ^†^ = 0.91)**									
1	Your ties (relationships) with your family have been …	0.50	1	5	3.19	1.15	−0.33	−0.48	−0.22	0.69
8	A sense that you are not the only one having a tough time has been gained …	0.63	1	5	3.51	1.04	−0.32	−0.37	−0.02	0.60
2	Your ties (relationships) with your friends and peers have been …	0.46	1	5	3.00	1.18	−0.11	−0.64	0.14	0.47
4	The peace of mind that you can get help from healthcare/ welfare staff and volunteers during difficult times has been gained …	0.58	1	5	3.42	1.17	−0.25	−0.65	0.18	0.47
6	Your compassion and empathy for people have been …	0.79	1	5	3.64	.920	−0.44	0.30	0.15	0.46
3	Trustworthy friends or peers you would not have met if you did not have mental illness have been gained …	0.82	1	5	2.92	1.24	0.16	−0.84	0.12	0.44
7	A sense that you got to be able to tell your feelings to those around you has been gained …	0.76	1	5	3.00	1.10	0.09	−0.48	0.36	0.43
5	The opportunities to greet and speak to neighbors and people in the community have been …	0.91	1	5	3.03	1.17	−0.26	−0.65	0.19	0.41
	Eigenvalue								10.15	1.22
	% Variance explained								46.15	5.55
	Cumulative % variance explained								46.15	51.70

^†^ Intraclass correlation coefficient (ICCs) and weighted kappas were calculated using the data of 19 respondents.

**Table 3:** Pearson’s (Spearman’s) correlation coefficients of the total benefit finding questionnaire (BFQ) score and scores of each factor with related scales (*N* = 265).

	Total	Factor 1	Factor 2
Related Scales	*r* (*ρ*) (*n*)	*r* (*ρ*) (*n*)	*r* (*ρ*) (*n*)
Recovery Assessment Scale (RAS)	0.84 ** (248)	0.86 ** (248)	0.67 ** (248)
Self-Identified Stage of Recovery Part-A ^†^ (SISR-A)	0.47 ** (261)	0.49 ** (261)	0.49 ** (261)
Self-Identified Stage of Recovery Part-B (SISR-B)	0.72 ** (263)	0.74 ** (263)	0.57 ** (263)
The Herth Hope Index (HHI)	0.72 ** (247)	0.75 ** (247)	0.57 ** (247)
SF-8 Mental Component Summary score	0.23 ** (256)	0.26 ** (256)	0.14 * (256)
SF-8 Physical Component Summary score	0.19 ** (256)	0.22 ** (256)	0.13 * (256)

^†^ Spearman’s correlation coefficients (*ρ*) were calculated. * *p* < 0.05. ** *p* < 0.01. The number of subjects varied because of missing responses for each related scale.

## Supplementary Materials

The following are available online at https://www.mdpi.com/2227-9032/8/3/303/s1, File 1: Benefit Finding Questionnaire (BFQ) (English), File 2: Benefit Finding Questionnaire (BFQ) (Japanese).
