# Supplementary material for: The Benefit Finding Questionnaire (BFQ): Scale Development, Validation, and Its Psychometric Properties Among People with Mental Illness
Source: Healthcare (Basel). 2020 Aug 26;8(3):303. doi: 10.3390/healthcare8030303 (PMC7551197; doi:10.3390/healthcare8030303)
Supplement: Supplementary file 1 [file healthcare-08-00303-s001.zip › Appendix A-English.pdf]

Appendix A. Benefit Finding Questionnaire (BFQ) (English)

Many people who have suffered an illness experience various changes. They may experience difficulties or feel that they have lost something. While coping with these changes, they may also feel that they have gained or learnt something. Compared with when you began suffering from (mental) illness, how do you think you have changed with respect to the following aspects?

Please circle the answer you feel is the most appropriate on a scale of 1–5.

1	Your ties (relationships) with your family have been<br>1_____2_____3_____4_____5<br>weakened relatively weakened neither relatively strengthened strengthened
2	Your ties (relationships) with your friends and peers have been<br>1_____2_____3_____4_____5<br>weakened relatively weakened neither relatively strengthened strengthened
3	Trustworthy friends or peers you would not have met if you did not have (mental) illness have been gained<br>1_____2_____3_____4_____5<br>none little somewhat quite a lot a lot
4	The peace of mind that you can get help from healthcare/ welfare staff and volunteers during difficult times has been gained<br>1_____2_____3_____4_____5<br>none little somewhat quite a lot a lot
5	The opportunities to greet and speak to neighbors and people in the community have been<br>1_____2_____3_____4_____5<br>decreased relatively decreased neither relatively increased increased
6	Your compassion and empathy for people have been<br>1_____2_____3_____4_____5<br>weakened relatively weakened neither relatively strengthened strengthened
7	A sense that you got to be able to tell your feelings to those around you has been gained<br>1_____2_____3_____4_____5<br>none little somewhat quite a lot a lot
8	A sense that you are not the only one having a tough time has been gained<br>1_____2_____3_____4_____5<br>none little somewhat quite a lot a lot
9	A feeling of reassurance knowing people who have similar illnesses or difficulties has been gained<br>1_____2_____3_____4_____5<br>none little somewhat quite a lot a lot
10	A feeling that you cherish yourself have been<br>1_____2_____3_____4_____5<br>weakened relatively weakened neither relatively strengthened strengthened

Compared with when you began suffering from (mental) illness, how do you think you have changed with respect to the following aspects?	
11	A sense that you are capable of facing your life with illness has been gained<br>1____2____3____4____5<br>none / little / somewhat / quite a lot / a lot
12	A feeling that you got to focus on things you can do rather than things you cannot do has been gained<br>1____2____3____4____5<br>none / little / somewhat / quite a lot / a lot
13	A sense that you would live by your own values rather than comparing yourself to others has been gained<br>1____2____3____4____5<br>none / little / somewhat / quite a lot / a lot
14	A feeling that you are happy to be alive has been<br>1____2____3____4____5<br>weakened / relatively weakened / neither / relatively strengthened / strengthened
15	Finding happiness in small things has become<br>1____2____3____4____5<br>difficult / relatively difficult / neither / relatively easy / easy
16	New something to live for or enjoyment in life has been gained<br>1____2____3____4____5<br>none / little / somewhat / quite a lot / a lot
17	A feeling that you are willing to begin something on your own has been<br>1____2____3____4____5<br>weakened / relatively weakened / neither / relatively strengthened / strengthened
18	Your mental strength has been<br>1____2____3____4____5<br>weakened / relatively weakened / neither / relatively strengthened / strengthened
19	A sense that you have become capable of feeling your hope and future has been gained<br>1____2____3____4____5<br>none / little / somewhat / quite a lot / a lot
20	To work toward a healthy lifestyle that suits you has become<br>1____2____3____4____5<br>difficult / relatively difficult / neither / relatively easy / easy
21	A feeling that you want to be of help to others and society has been<br>1____2____3____4____5<br>weakened / relatively weakened / neither / relatively strengthened / strengthened

Note:

This English version is a simple translation from the original Japanese scale, without translation and back translation procedure.

We wrote "mental" in brackets in the introductory sentences and the sentence of #3.
