# Supplementary material for: The Benefit Finding Questionnaire (BFQ): Scale Development, Validation, and Its Psychometric Properties Among People with Mental Illness
Source: Healthcare (Basel). 2020 Aug 26;8(3):303. doi: 10.3390/healthcare8030303 (PMC7551197; doi:10.3390/healthcare8030303)
Supplement: Supplementary file 1 [file healthcare-08-00303-s001.zip › Appendix B-Japanese.pdf]

Appendix B. Benefit Finding Questionnaire (BFQ) (Japanese)

病を経験した人は、様々な変化を感じるといわれています。困難を感じたり、何かを失ったと感じたりすることもあるかもしれません。また、そうした変化に対処することを通して、何かを得たり学んだりしたと感じることもあるかもしれません。

以下について、**(精神)の病になった当初と比べて、**今はどのように変化したと感じていますか。

１から５のうち、もっともあてはまると思うもの１つに〇をつけてください。

1	**家族との絆(関係)は**				
	1	2	3	4	5
	弱くなった	どちらかといえば弱くなった	どちらともいえない	どちらかといえば強くなった	強くなった
2	**友人や仲間との絆(関係)は**				
	1	2	3	4	5
	弱くなった	どちらかといえば弱くなった	どちらともいえない	どちらかといえば強くなった	強くなった
3	**(精神)の病になっていなければ得られなかったような、信頼できる友人や仲間は**				
	1	2	3	4	5
	得られていない	少ししか得られていない	やや得られた	かなり得られた	おおいに得られた
4	**困ったときには医療や福祉のスタッフやボランティアの人たちから助けを得られるという安心感は**				
	1	2	3	4	5
	得られていない	少ししか得られていない	やや得られた	かなり得られた	おおいに得られた
5	**近所や地域の人たちとあいさつしたり話したりする機会は**				
	1	2	3	4	5
	減った	どちらかといえば減った	どちらともいえない	どちらかといえば増えた	増えた
6	**人に対する気配りや人を思いやる気持ちは**				
	1	2	3	4	5
	弱くなった	どちらかといえば弱くなった	どちらともいえない	どちらかといえば強くなった	強くなった
7	**周囲の人に気持ちを伝えられるようになったという感覚は**				
	1	2	3	4	5
	得られていない	少ししか得られていない	やや得られた	かなり得られた	おおいに得られた
8	**自分だけが大変なわけではないという感覚は**				
	1	2	3	4	5
	得られていない	少ししか得られていない	やや得られた	かなり得られた	おおいに得られた
9	**同じような病や悩みをもつ人を知って心強いという感覚は**				
	1	2	3	4	5
	得られていない	少ししか得られていない	やや得られた	かなり得られた	おおいに得られた
10	**自分を大切にする気持ちは**				
	1	2	3	4	5
	弱くなった	どちらかといえば弱くなった	どちらともいえない	どちらかといえば強くなった	強くなった

以下について、**(精神)の病になった当初と比べて、**今はどのように変化したと感じていますか。	
11	**病のある自分の人生と向き合えているという感覚は**<br>1 得られていない　2 少ししか得られていない　3 やや得られた　4 かなり得られた　5 おおいに得られた
12	**できないことよりもできることに目を向けられるようになったという感覚は**<br>1 得られていない　2 少ししか得られていない　3 やや得られた　4 かなり得られた　5 おおいに得られた
13	**人と比べるよりは自分らしく生きていこうという感覚は**<br>1 得られていない　2 少ししか得られていない　3 やや得られた　4 かなり得られた　5 おおいに得られた
14	**生きていてよかったと思う気持ちは**<br>1 弱くなった　2 どちらかといえば弱くなった　3 どちらともいえない　4 どちらかといえば強くなった　5 強くなった
15	**小さな幸せを見つけることが**<br>1 難しくなった　2 どちらかといえば難しくなった　3 どちらともいえない　4 どちらかといえばできるようになった　5 できるようになった
16	**新しい生きがいや人生のたのしみは**<br>1 得られていない　2 少ししか得られていない　3 やや得られた　4 かなり得られた　5 おおいに得られた
17	**自分で何かを始めてみようという気持ちは**<br>1 弱くなった　2 どちらかといえば弱くなった　3 どちらともいえない　4 どちらかといえば強くなった　5 強くなった
18	**あなたの精神的な強さは**<br>1 弱くなった　2 どちらかといえば弱くなった　3 どちらともいえない　4 どちらかといえば強くなった　5 強くなった
19	**希望や未来を感じられるようになったという感覚は**<br>1 得られていない　2 少ししか得られていない　3 やや得られた　4 かなり得られた　5 おおいに得られた
20	**自分に合った健康的な生活スタイルにつとめることが**<br>1 できなくなった　2 どちらかといえばできなくなった　3 どちらともいえない　4 どちらかといえばできるようになった　5 できるようになった
21	**人や社会のために役に立ちたいという気持ちは**<br>1 弱くなった　2 どちらかといえば弱くなった　3 どちらともいえない　4 どちらかといえば強くなった　5 強くなった

Note: 本研究では、教示文と No.3 の(　)内に「精神」と入れています
